# “I need to get back to a normal life”: using the core DSM-5 Cultural Formulation Interview to explore existential themes shared by adolescents in specialized mental healthcare in Norway

**DOI:** 10.3389/fpsyg.2025.1652189

**Published:** 2025-11-12

**Authors:** Nina Therese Øversveen Svamo, Sigrid Helene Kjørven Haug, Valerie DeMarinis

**Affiliations:** 1 Research Center for Existential Health, Innlandet Hospital Trust, Brumunddal, Norway; 2 Faculty of Social and Health Sciences, University of Inland Norway, Lillehammer, Norway; 3 Department of Public Health and Clinical Medicine, Umeå University, Umeå, Sweden

**Keywords:** Cultural Formulation Interview, adolescent, mental healthcare, clinical setting, existential, meaning-making, narratives

## Abstract

**Background:**

Despite the high prevalence of mental health challenges among adolescents, often they do not receive sufficient support in mental healthcare. Therefore, person-centered care (PCC) is essential for accessing adolescents' needs, resources and preferences. The DSM-5 core Cultural Formulation Interview (CFI) is a PCC tool designed to elicit patients' narratives about illness and health. However, knowledge about its application with adolescents remains limited. The existential dimension, an implicit content area of the CFI, is an important and often overlooked dimension in mental healthcare. This study aimed to explore the CFI's contribution to identifying the salient existential themes shared by adolescents while receiving treatment in a specialized mental healthcare inpatient unit in Norway. The study is framed by culturally-informed, multi-dimensional and holistic understandings of health and illness, where the existential dimension can play a fundamental role in PCC treatment planning and treatment process.

**Methods:**

This qualitative study is part of a larger mixed-methods project. Six consecutive adolescents aged 14–17 years, with various mental health problems, were interviewed by trained clinicians, using the semi-structured CFI interview. The interviews were analyzed with inductive content analysis.

**Results:**

Four main categories emerged through the analysis: (1) Striving to achieve a normal everyday life, (2) Seeking supportive understanding from family and clinicians, (3) Dealing with adverse childhood experiences, and (4) Struggling with ongoing challenges and future uncertainty.

**Conclusion:**

This study highlights the potential of the CFI as a narrative tool to enhance understanding of adolescents' existential concerns and needs in mental healthcare treatment. Their narratives concerned multi-dimensional and interacting aspects of illness and health (biological-physical, psychological, social, ecological, and existential aspects) of relevance to treatment planning and the treatment process. Notably, a functional understanding of the existential dimension, designed for clinical contexts, provided a deeper and more nuanced perspective on how existential issues influence decision-making and coping with everyday challenges. The findings indicate the importance of including existential themes in mental healthcare and treatment for adolescents. The qualitative data in this study were derived from a larger mixed-methods project designed to test the efficacy of the CFI in different clinical contexts.

## Introduction

In 2015, all United Nations member states committed to the 2030 Agenda for Sustainable Development, which outlines 17 Sustainable Development Goals (SDGs) to address global challenges such as poverty, inequality, climate change, and health (United Nations Sustainable Development Goals, 2015). Among these, SDG 3 aims to “ensure healthy lives and promote wellbeing for all at all ages.” This goal is particularly relevant for adolescents, defined by the World Health Organization (WHO) as individuals aged 10–19 years, because early interventions can address emerging concerns and help young people develop the resources needed to live healthy and fulfilling lives (World Health Organization, 2018). The United Nations Secretary General's Global Strategy for Women's, Children's and Adolescents' Health (2016–2030), reinforces this priority, aiming to “ensure health and wellbeing for every woman, child and adolescent” within the broader SDG framework (United Nations Secretary-General, 2015). Mental health has been identified by the WHO as a particularly urgent concern for this age group, with mental health problems representing the leading health burden among adolescents and accounting for 13% of the global disease burden (World Health Organization, 2024). Poor mental health during adolescence can have lasting negative effects on health, education, employment, family and social life, and overall wellbeing (Westberg et al., 2022). Nevertheless, a large proportion of adolescents experiencing such challenges do not receive the necessary support (Radez et al., 2021).

For adolescents receiving help in specialized mental healthcare, person-centered care (PCC) is essential to enhance their mental health outcomes (World Health Organization, 2012). PCC is a holistic approach that places the unique needs and preferences of each individual at the center of healthcare (Morgan and Yoder, 2012). A recent scoping review on PCC, focusing on adolescents' understanding of self-engagement in treatment (Svamo et al., 2024), found that adolescents especially value a strong therapeutic alliance, and active involvement in all stages of therapy. Adolescents value feeling understood and included in the therapeutic process (Stige et al., 2021), and poor engagement is often linked to alliance ruptures and epistemic mistrust (Cirasola et al., 2024). Active involvement in treatment requires healthcare professionals to be attentive to adolescents' needs and preferences (Svamo et al., 2024) by listening to what is important for them.

While PCC is increasingly recognized as essential in adolescent mental healthcare (Stige et al., 2021; Cirasola et al., 2024; Svamo et al., 2024), significant gaps remain in addressing adolescents' existential concerns within mental healthcare (Løkke, 2025; Menzies et al., 2025). Existential concerns, such as meaning-making, identity, and worldview, are critical to adolescents' development but are often overlooked in clinical practice (Ulland and DeMarinis, 2014). Unresolved existential struggles, such as loneliness, fear of death, and alienation, can exacerbate mental health vulnerabilities, particularly among young women (Lloyd et al., 2016). To better address these needs, knowledge of existential concerns and a sensitivity to adolescents' personal needs are essential.

The DSM-5 core Cultural Formulation Interview (CFI) is a PCC-based clinical tool designed to strengthen patients' voices in treatment and care through an holistic exploration of their cultural and personal health narratives (American Psychiatric Association, 2013, 2022). Most studies on the CFI have focused on adult minority populations, leaving its applicability with adolescents in both majority and minority populations underexplored (Kirmayer, 2015; Rousseau and Guzder, 2016; Lewis-Fernández et al., 2020). To begin to address this gap, this study explores the DSM-5 CFI's contribution to identifying the salient existential themes shared by majority-population adolescents while receiving treatment in a specialized mental healthcare inpatient unit in Norway. The study was framed by a culturally-informed, multi-dimensional and holistic understandings of health and illness, where the existential dimension, in a multi-dimensional clinical framework, plays a fundamental role in treatment planning (Kleinman, 1980; DeMarinis, 2003, 2006, 2008).

### Conceptual clarifications

Kleinman's work in medical anthropology and psychiatry has been pivotal in developing concepts and clinical models to strengthen PCC in clinical settings (Kleinman, 2016; Lewis-Fernández et al., 2016). His contributions have also served as a basis for the development of the CFI (Kleinman, 2016; Kirmayer et al., 2023). Kleinman introduced the term “illness narrative,” defining it as “a story the patient tells, and significant others retell, to give coherence to the distinctive events and long-term course of suffering” (Kleinman, 1988, p. 49). Through illness narratives, patients give voice to their multi-level experiences of suffering, aiming to better understand and communicate the meaning of their illness and its context (Kleinman and Benson, 2006). These narratives illuminate the meaning of care, not only for practitioners but also for patients and their families. Building on Kleinman's understanding of illness narratives, Kirmayer et al. (2023), emphasized a broader understanding of patients' stories as “narratives,” meaning “the experience of health and illness, framing symptoms and giving them meaning, guiding, coping, help-seeking, and the response to interventions” (Kirmayer et al., 2023, p. 236). Kirmayer et al. (2023) further highlighted that narratives function as fundamental forms of communication, not only conveying information but also organizing experience, thought and action.

This broader perspective highlights the dynamic and relational nature of narrative processes, which guide patients' coping strategies and clinicians' capacity to engage in collaborative care. Narrative methods provide a platform for active patient participation and help clinicians develop listening skills, ultimately enriching their understanding of health problems (Kirmayer et al., 2023). In line with this perspective, narratives encompass multi-dimensional aspects of both illness and health, which allows for further shaping the treatment process in holistic and meaningful ways.

The present study builds on Kleinman's cultural and multi-dimensional model of healthcare systems (Kleinman, 1980, p. 27–45), which encompasses various dimensions of health. These dimensions (biological-physical, psychological, social, ecological, and symbolic) have been further developed and adapted to Scandinavian healthcare contexts by DeMarinis (2003, 2006, 2008). DeMarinis expanded Kleinman's framework by adapting the symbolic dimension in the original model to the existential dimension, resulting in five interacting dimensions: biological-physical, psychological, social, ecological, and existential (DeMarinis, 2003, p. 44–46). The existential dimension includes “worldview conception, life approach, decision-making structure, ways of relating, ways of understanding, and activities or expressions of symbolic significance such as ways of making meaning” (DeMarinis, 2003, p. 45).

A core element of this perspective is the recognition that every individual has a dynamic narrative that informs the dimensions of illness and health. Central to that narrative is an existential dimension, which may be expressed in a variety of ways. The existential dimension is explored from a functional perspective in the clinical context, focusing on the actual roles and effects this type of information has for the individual in approaching decisions and addressing everyday challenges. Often this information contains resources but also problematic or unclear elements that need to be understood and addressed. Insight into the function of the existential dimension's information is considered critical for understanding how expressions and experiences of health and illness are constructed in daily life. Furthermore, this dimension interacts with and influences the other dimensions (biological-physical, psychological, social, and ecological) by shaping adjustments, interpretations, and behaviors related to illness and health experiences (DeMarinis, 2003, p. 45–46).

Conceptualizations of the existential dimension, using a variety of terminology, have been applied in health-related research in Scandinavia, primarily focusing on adult populations across various contexts. Examples include patients at risk of suicide in specialized mental healthcare (Søberg et al., 2022), older people with incurable cancer in specialized palliative care (Haug et al., 2015, 2016), Christian congregation members with mental illnesses (Lilja et al., 2016), and callers to a diaconal suicide prevention crisis line (Vattø et al., 2020). These studies show that integrating the existential dimension into PCC is essential for enhancing resilience and addressing individual needs in various clinical settings. Additionally, they highlight the importance of understanding how existential, psychological, and social factors interplay within specific cultural contexts, emphasizing the need for more personalized approaches and expanded clinical training in this domain.

In Scandinavia, studies highlight the importance of accessing and addressing existential themes during adolescence across different contexts and theoretical frameworks. Garnow et al. (2022, 2024b) explore how adolescents in Swedish upper-secondary school experience existential loneliness, being defined as a fundamental sense of being ≪separated from others and the wider world≫ (2022). Existential loneliness is experienced as an immense, consuming, and challenging to manage alone (Garnow et al., 2022, 2024b). A recent study by Garnow et al. (2024a) emphasize the importance of sensitivity to adolescents' individual needs, including existential loneliness. The study conclude that fostering supportive relationships can help adolescents cope with existential loneliness, promote wellbeing, and personal growth. In an outpatient mental healthcare clinic for children and adolescents in Norway, Olstad et al. (2023) explore how adolescents aged 14–18 with developmental trauma in experience meaning in life based on Schnell (2009, 2021). The results show that meaning is tied to coherence, belonging, and intrinsic values, with sources such as relationships, routines, and personal achievements. A related study by Olstad et al. (2022) with therapists, working in the same outpatient setting, examines how meaning in life is understood and addressed in adolescents with developmental trauma. While therapists acknowledge the relevance of these issues they report limited competence in systematically addressing them (Olstad et al., 2022). Regarding research applying DeMarinis' existential dimension framework (DeMarinis, 2003, p. 45) in mental healthcare for adolescents and younger adults in Scandinavia, two studies have been identified. The first study collects qualitative interviews with therapists working in a mental health outpatient clinic for adolescents in Norway (Ulland and DeMarinis, 2014). It shows that therapists prefer a broad approach to the existential domain to capture its various forms of expression. The study also identifies shortcomings in clinical education and training for working with clients in this domain. The second study, using mixed-methods, explore the relationship between existential meaning-making and emotion regulation among young women aged 19–24 in mental healthcare in Sweden (Lloyd et al., 2016). The study reveal that frequent relational losses and disruptions heightened the participants' psychological and existential vulnerabilities, leading to challenges such as fear of death, loneliness, and alienation. It recommends addressing everyday existential concerns during treatment to provide a deeper understanding of the presenting problems, to identify potential resources and to clarify therapeutic expectations (Lloyd et al., 2016).

### The DSM-5 Cultural Formulation Interview (CFI)

The purpose of the CFI is to allow the patient's voice regarding illness, health, and care to assume a primary function in the therapeutic process (Lewis-Fernández et al., 2016). This approach is grounded in the DSM-5's definition of culture, which encompasses the existential dimension (American Psychiatric Association, 2013, 2022):

Culture refers to systems of knowledge, concepts, values, norms, and practices that are learned and transmitted across generations. Culture includes language, religion and spirituality, family structures, life-cycle stages, ceremonial rituals, customs, and ways of understanding health and illness, as well as moral, political, economic, and legal systems (American Psychiatric Association, 2022, p. 860).

This definition reflects a broad and dynamic understanding of culture. Individuals are influenced by multiple cultural factors, which they use to shape their identities and make sense of the world. This process of meaning-making emerges from developmental and everyday social experiences within specific contexts, including healthcare, which may vary significantly between individuals (American Psychiatric Association, 2022).

The core CFI is described by Kirmayer as a holistic model for eliciting patient narratives (Kirmayer et al., 2023). The interview consists of 16 open-ended questions organized into four main domains: “Cultural definition of the problem,” “Cultural perceptions of cause, context, and support,” “Cultural factors affecting self-coping and past help-seeking,” and “Cultural factors affecting current help-seeking” (Aggarwal et al., 2016a). Additionally, 12 supplementary modules are available to address specific topics relevant to various populations, such as school-age children and adolescents, to explore the cultural dynamics between home, school, and peer groups (Rousseau and Guzder, 2016). For this study, only the core CFI with its 16 questions was used. The interview was translated by a multi-disciplinary healthcare and research team into Norwegian in 2015 (Migration Health Norwegian National Advisory Unit on Concurrent Substance Abuse Mental Health Disorders, 2015), funded by the Norwegian Directorate of Health. It was subsequently integrated into the “National Patient Pathway” in 2018 [Helsedirektoratet (Norwegian Directorate of Health), 2018], targeting children and adolescents in specialized mental healthcare. A central aim of this integration was to enhance patient involvement and satisfaction. In this context, the question “What is important to you?” is considered fundamental for improving care quality and fostering meaningful conversations between patients, caregivers, families, and healthcare providers (Norwegian Institute of Public Health, 2024). This aligns closely with the purpose of the CFI.

Most studies on the CFI have focused primarily on adult minority populations in mental healthcare contexts (Wallin et al., 2024). Research indicates that the CFI facilitates communication and strengthens the therapeutic alliance between patients and clinicians (Aggarwal et al., 2015; Muralidharan et al., 2017). Additionally, the CFI has been shown to effectively elicit social determinants of mental health (Aggarwal et al., 2023). Certain areas for refinement have been identified, such as addressing socio-structural determinants of health (Aggarwal et al., 2023) and the complexity of operationalizing conceptions of identity in clinical settings (Wallin et al., 2020; Lindberg et al., 2021, 2022). A qualitative study conducted with native Swedish-speaking patients (Wallin et al., 2024) suggests that the CFI is also a valuable tool for exploring cultural and social factors in majority populations. The study recommends further adaptations to improve the tool's applicability to these groups (Wallin et al., 2024). A study from the Netherlands (Silvius et al., 2024), focusing on adults in a large mental health organization, emphasizes the importance of clinician training in the implementation of the CFI. Findings indicate that clinicians with higher cultural competence are more likely to implement the CFI effectively, whereas a lack of mandatory training limits its broader adoption (Lewis-Fernández et al., 2020; Jones-Lavallée et al., 2022).

Regarding CFI research on adolescents in mental healthcare (Shem-Tov et al., 2018; Arshad et al., 2022; Sanchez et al., 2022a), a case study (Sanchez et al., 2022a) as well as a clinical article using case illustrations (Arshad et al., 2022) both from the United States and a case study from Israel (Shem-Tov et al., 2018) were identified, all of which focus on diverse groups of minority patients. The case study from the US (Sanchez et al., 2022a) is based on a previous randomized controlled trial (Sanchez et al., 2022b) examining the use of the CFI for parents in family-based mental healthcare. The findings show that families feel more understood by their therapist, leading to improved treatment engagement and response. In the case study (Sanchez et al., 2022a), treatment of a 17-year-old girl of minority background is integrated with a case conceptualization model based on cultural formulations in the CFI. The findings highlight how the CFI redistributes the power dynamic in therapy by valuing the patient's perspective, preferences, and experiences (Sanchez et al., 2022a). The Israeli case study (Shem-Tov et al., 2018) uses a culturally-sensitive diagnostic model based on the CFI with two Ethiopian adolescents with eating disorders living in Israel. The findings show that the CFI facilitates treatment breakthrough, helping the therapist to understand the cultural and familial meanings behind the adolescents' symptoms, such as stomachache (Shem-Tov et al., 2018). The clinical article from the United States (Arshad et al., 2022) uses case illustrations of three adolescents, aged 15–18, to demonstrate the clinical utility of the CFI. The findings suggest that cultural assessment should be integrated into all clinical encounters but is particularly beneficial in complex diagnostic situations, when patient engagement and adherence are limited, or when there is disagreement between clinician and patient (Arshad et al., 2022). Despite these promising findings, research on the use of the CFI with adolescents in mental healthcare remains limited, especially in majority populations (Kirmayer, 2015; Rousseau and Guzder, 2016; Lewis-Fernández et al., 2020; Jones-Lavallée et al., 2022).

### Research question

The present study is seen as a contribution to filling this gap by addressing the research question:

What are the salient existential themes adolescents in specialized mental healthcare treatment in Norway share when interviewed with the core DSM-5 Cultural Formulation Interview?

## Materials and methods

### Study design

The present study was a sub-study in a mix-methods study designed to test the efficacy of the CFI in a specialized mental healthcare inpatient unit for adolescents. The design was derived from a larger mix-methods project, focusing on efficacy testing of the CFI in different clinical contexts. Efficacy was operationalized as feasibility, acceptability, and clinical utility, in line with the CFI international field trials (Lewis-Fernandez et al., 2017). The structured methodological format included documentation and analysis of different stages in the research process such as: CFI training with clinicians, a fidelity assessment of clinicians' administration of the core CFI, interviews with adolescents and clinicians, and debriefing interviews with both groups. The format was further adapted by the third author (VDM), encompassing cultural analysis of the clinical site, and separate efficacy evaluations by clinicians and adolescents. More specifically, this involved interviews with clinicians at different stages of the research process, and interviews with adolescents before completion of treatment. In this way, the core CFI (T1-core CFI) was tested for efficacy at several points (T2, T3) in time during the treatment process. This was described as the CFI process.

This qualitative study has focused solely on the interviews with the core CFI (T1-core CFI) to highlight the adolescents' perspectives and narratives. Clinical utility refers to the usefulness and added value of the CFI in eliciting meaningful narratives and facilitating a person-centered understanding of the adolescents' experiences. We have applied an inductive and exploratory approach to understand adolescents' voices on what they found important while receiving treatment in a specialized mental healthcare inpatient unit. The data consists of the core CFI conducted with six consecutive adolescents during the period from September 2020 to February 2021. The content of the material consists of illness and health narratives, in line with the intention of the CFI (Kirmayer et al., 2023). Inductive content analysis was the analytic strategy adopted (Elo and Kyngäs, 2008).

### Context

The context of the study is a facility providing treatment for adolescents aged 12-18 with moderate to severe mental health disorders and a high rate of comorbidity. The inpatient unit receives adolescents from across a wide geographical area, including both urban centers and smaller rural communities. The inpatient unit specializes in treating serious eating disorders. Adolescents with other complex problems such as school truancy, self-harm, and severe depression are also included for treatment. With its six beds, the inpatient unit offers treatment stays of 14 days, 6 weeks, or 3 months. The clinicians, i.e., doctors, psychologists, social workers, and nurses, work together in teams. A separate school unit is adjacent to the inpatient unit. Throughout the adolescents' treatment, the unit collaborates with families, schools, primary healthcare contexts, and referring authorities.

### Sample and procedure

The inclusion criteria were determined in consultation with the clinicians, with the initial aim of including adolescents aged 16–18 years old. Since most of the patients were 12–16 years old at the time of inclusion, the CFI was tested on three adolescents aged 14–15, resulting in positive responses from both adolescents and clinicians. Consequently, the age range was adjusted to 14–18 years. Following the methodological format of the larger project testing the efficacy of the core CFI and following the CFI process through the treatment process for each patient in the different clinical contexts, six consecutive patients in each clinical context fulfilling the context-specific inclusion criteria and formally consenting to being in the study were included. Therefore, six consecutive adolescent patients were included in the study. Potential participants needed to be assessed as cognitively and emotionally suitable for the core CFI and CFI process by clinicians responsible for the treatment. Exclusion criteria were: lack of capacity to consent, ongoing crisis or recent traumatic event, psychosis, severe depression, serious suicide risk, and serious ongoing substance abuse. The sample consisted of five girls and one boy aged 14–17 years. Five were under 16 years of age. The adolescents came from surrounding towns and rural communities near to natural areas such as mountains, lakes and forests. Adjustments to the timing of administering the core CFI were necessitated by challenges in recruiting patients, primarily stemming from the situation at the inpatient unit, including factors such as staff illness and COVID-19. Although the core CFI is intended to be used with each patient as early in the process as possible, it was administered at different time points, with two interviews conducted in the first week, one in the second week, two in the third week, and one in the fourth week. This was due to the need to find a suitable time for both patient and clinician. Notably, the interview in the fourth week was for an adolescent admitted to the clinic for a second time. This contributed to the variability in the timing and in the second admittance case made the core CFI a means of reacquainting the clinic staff with the patient.

Gender, age, time of conducting the CFI during admission, and diagnostic data from the psychiatric summary in the medical record are provided in Table 1. For anonymity, the diagnostic data only indicate the main mental and behavioral disorders. According to medical records, all participants self-identified as ethnic Norwegians. The core CFI (T1) was conducted face-to-face in a suitable room in the inpatient unit, and lasted from 22 to 59 min, with a mean of 36 min. The six interviews were audio recorded and transcribed verbatim into Norwegian by the first author. The transcriptions were then imported to a secure research server at the hospital.

**Table 1:** Characteristics of the sample.

**The core CFI**	**CFI 1**	**CFI 2**	**CFI 3**	**CFI 4**	**CFI 5**	**CFI 6**
Patient gender/age	F 15	M 17	F 15	F 15	F 14	F 15
Mental and behavioral disorders (ICD 10)	F50.0 Anorexia nervosa	F32.1 Moderate depression episode	F50.0 Anorexia nervosa	F50.0 Anorexia nervosa	F90 Hyperkinetic disorders (ADHD)	F90 Hyperkinetic disorders (ADHD)
Time of conducting the CFI during admission	Fourth week^*^	First week	Third week	Third week	Second week	First week

^*^This was in the second treatment period for the patient.

### The core CFI—adherence and competence

The core CFI (Lewis-Fernández et al., 2016) from DSM-5 (American Psychiatric Association, 1994, 2022) is a validated tool designed to elicit patients' perspectives on illness, health, and care in relation to their cultural context. The interview consists of 16 open-ended questions organized into four main domains (Aggarwal et al., 2016a), as described in the introduction.

Three clinical staff members received specific CFI training to conduct the core CFI for adolescents. The training was designed and adapted to this mental health context by the research teams' psychologist and mental health clinician, and inspired by the design used by Aggarwal et al. (2016b) utilized in the DSM-5 field trials (Lewis-Fernandez et al., 2017), aiming to apply the core CFI in a person-centered manner. The training included cultural competence as a core component and consisted of three elements: (a) Preparatory work, (b) One-day training session with behavioral simulations, and (c) Ongoing consultation and feedback (part of the CFI process) throughout the treatment process.

a) Preparatory work: Clinicians engaged in self-reflection by watching a video of a core CFI role play, tested the CFI on themselves and prepared an adolescent case for the behavioral simulation session.
b) One-day training session: Patient cases were used in behavioral simulation sessions, where clinicians alternated roles as both interviewer and adolescent. During the training, the authors emphasized the importance of probing, asking for examples from daily life, and following the order of the questions (1–16). Both the self-testing analysis and the case-based simulation sessions were facilitated by the authors (VDM and SHKH).

As part of the training, the clinical group reviewed and discussed each question in the CFI in relation to the adolescent patient group. These discussions led to several modifications to the original CFI, including adjustments to wording, simplifications, and cultural adaptations to better fit the clinical and linguistic context (DeSilva et al., 2018; Wallin et al., 2020). For example, the Norwegian translation of the term “spiritual reason” required clarification using several synonyms to ensure comprehension for participants.

Additional words and phrases were incorporated to further align the CFI with the clinical context, and four sub-questions were added in collaboration with the clinicians. A complete overview of the DSM-5 core CFI, along with all adjustments made for use in an adolescent mental health clinic in Norway, is presented in Table 2. In this table, text that has been reformulated from the original DSM-5 core Cultural Formulation Interview (American Psychiatric Association, 2013, 2022) is marked with a strikethrough, while newly added text is highlighted in bold. These adaptations were made to enhance the cultural and contextual relevance of the CFI for adolescents in Norway. Further explanations for the rationale for these adaptions are provided in the Supplementary file.

c) Ongoing consultation and feedback: The authors provided support, answering questions and offering the clinical team feedback on the CFI process with the six participants.

**Table 2:** The DSM-5 core CFI with adjustments for use in an adolescent mental health clinic in Norway.

**Question**	**Instructions for clinicians**
	INTRODUCTION FOR THE INDIVIDUAL: I would like to understand the problems that bring you here so that I can help you more effectively. I want to know about your experience and ideas. I will ask some questions about what is going on and how you are dealing with it. Please remember there are no right or wrong answers The point is to listen to what you say. If something important or difficult comes up, we will return to it after the interview
**Cultural definition of the problem**	
1.	What brings you here **at the inpatient unit**? IF INDIVIDUAL GIVES FEW DETAILS OR ONLY MENTIONS SYMPTOMS OR A MEDICAL DIAGNOSIS, PROBE: People often understand their problems in their own way, which may be similar to or different from how doctors describe the problem How would you describe your problem?
2.	Sometimes people have different ways of describing their problem to their family, friends, or others in their community. How would you describe your problem to them?
3.	What troubles you most about your problem? **How do you feel it in your body?**
**Cultural perceptions of cause, context, and support**	
4.	Why do you think this is happening to you? What do you think are the causes of your [PROBLEM]? PROMPT FURTHER IF REQUIRED: Some people may explain their problem as the result of bad things that happen in their life, problems with others, a physical illness, a **synonym**, or many other causes
5.	What do others in your family, your friends, or others in your community think is causing your [PROBLEM]?
6.	Are there any kinds of support that make your [PROBLEM] **synonym**, such as support from family, friends, **contacts on social media** or others?
7.	Is it **synonym** that make your [PROBLEM] worse, such as difficulties with money, or family problems**, bullying or school**?
8.	Sometimes, aspects of people's background or identity can make their [PROBLEM] better or worse. By **synonym**, I mean, for example, the communities you belong to, the languages you speak, where you or your family are from, your race or ethnic background, your gender or sexual orientation, or your faith or religion For you, what are the most important aspects of your **upbringing? What gives you most meaning in life? What makes you experience life as empty or meaningless?**
9.	Are there any aspects of your **synonym** that make a difference to your [PROBLEM]?
10.	Are there any aspects of your **synonym** that are causing other concerns or difficulties for you?
**Cultural factors affecting self-coping and past help seeking**	
11.	Sometimes people have various ways of dealing with problems like [PROBLEM]. What have you done on your own to cope with your [PROBLEM]?
12.	Often, people look for help from many different sources, including different kinds of doctors, **psychologists**, helpers, or healers. In the past, what kinds of treatment, help, advice, or healing have you sought for your [PROBLEM]? **What kind of help have you sought elsewhere, such as at school, from friends, relatives, managers and social media**? PROBE IF DOES NOT DESCRIBE USEFULNESS OF HELP RECEIVED: What types of help or treatment were most useful? Not useful?
13.	Has anything prevented you from getting the help you need? PROBE AS NEEDED: For example, money, work or family commitments, stigma or discrimination, **bullying, challenges in the family, or various help services that have not understood you**?
**Cultural factors affecting current help seeking**	
14.	Now let's talk some more about the help you need. What kinds of help do you think would be most useful to you at this time for your [PROBLEM]?
15.	Are there other kinds of help that your family, friends, or other people have suggested would be helpful **to you as you are now**?
16.	Sometimes **we who work at the inpatient unit and the adolescents who are here can** misunderstand each other because they come from different backgrounds or have different expectations Have you been concerned about this and is there anything that we can do to provide you with the care you need?

### Analysis

Initially, a deductive approach was applied, guided by the structure of the CFI and its four core domains (Aggarwal et al., 2016a). However, it became evident that this approach did not fully capture the richness of adolescents' narratives, as their voices and personal contexts were not sufficiently represented. Consequently, an inductive approach was employed to allow the narratives to shape the analysis more naturally. To facilitate this, inductive content analysis was applied, as described by Elo and Kyngäs (2008). In this type of analysis, the text is sorted into smaller (but replicable and valid) inference categories, keeping the content as congruent as possible throughout the analysis (Elo and Kyngäs, 2008; Krippendorff, 2018). In addition, both manifest and latent content can be explored (Graneheim and Lundman, 2004; Graneheim et al., 2017). Since the material consisted of narratives, the latent meaning was selected in order to analyze the underlying meaning of the narratives (Graneheim and Lundman, 2004). They were mostly structured around a specific experience, related to difficult emotions, interactions with others, and intrapsychic processes. Table 3 illustrates the analysis process, moving from specific observations to a general statement (Elo and Kyngäs, 2008). The narratives were grouped into sub-categories (level 1), then into higher order categories as generic categories (level 2), and finally into main categories (level 3).

**Table 3:** Examples of the analysis process of the study, moving from short narrative, sub-category, and generic category to main category (Elo and Kyngäs, 2008).

**Short narrative**	**Sub-category (level 1)**	**Generic category (level 2)**	**Main category (level 3)**
I know I need food, but it's not that simple. You can't just tell someone who's struggling with an eating disorder to start eating. Or tell someone who's dealing with depression to just be happy. Or someone with anxiety to not be afraid. It doesn't work that way	I know I need food, but you can't just tell someone to change—it doesn't work that way	The importance of support and understanding from family and clinicians	Seeking supportive understanding from family and clinicians

The inductive content analysis consists of three phases: Preparation, organization, and reporting (Elo and Kyngäs, 2008):

1. The preparation phase: This phase focus on defining the object of study and becoming familiar with the text (Elo and Kyngäs, 2008). In the present study, the six core CFIs were reviewed line by line, identifying central themes
2. The organization phase: This phase involve five steps: Open coding, coding sheets, grouping, categorization, and abstraction (Elo and Kyngäs, 2008). The analysis process was iterative, with frequent cross-referencing to the questions in the CFI to ensure that the findings were grounded in the interview structure.
  2.1 Open coding: NVivo for Windows (R1.6), a computer-assisted qualitative data analysis software package, was used as a sorting tool, providing an overview of the data with initial categories. These narratives were then sorted into initial categories within the four core CFI domains (Aggarwal et al., 2016a). During this step, specific attention was paid to linking each narrative to the corresponding CFI question. This step revealed how adolescents' responses often spanned multiple questions, demonstrating the dynamic nature of the CFI. For example, narratives introduced in response to Question 4 (about the causes of the problem) were often elaborated on in response to Question 8 (about background and identity). Another example was that narratives about family relationships often appeared in responses to multiple questions, such as Question 8 (about background and identity) and Question 13 (about barriers to care). The short narratives formed the basis for the headings in the second step.
  2.2 Coding sheets: A coding sheet was created to list headings derived from the narratives in order to get an overview of the content. According to Elo and Kyngäs (2008), creating categories is about “classifying data that are belonging to a particular group.” In this step, categories were understood as groups of narratives that belonged thematically together. The group of narratives were placed under each of the four CFI domains. Examples of headings were Meaningful activities in daily life and Caring for siblings
  2.3 Grouping: Grouping is about reducing the number of categories by combining higher order headings with similar content (Elo and Kyngäs, 2008). As described in 2.1, the analysis in the present study revealed that the narratives evolved in different parts of the CFI since they were informed by the dynamic interplay between the CFI questions. Thus, the grouping was carried out across the four core CFI domains in terms of restructuring the identified narratives from the four CFI domains under the higher order headings.
  2.4 Categorization: This step includes condensing and grouping into preliminary categories to provide understanding and generate knowledge (Elo and Kyngäs, 2008). Examples of categories in this step were to be recognized by trusted people, and to recall difficult school experiences.
  2.5 Abstraction: The purpose in this step is to provide a more comprehensive understanding of the latent meaning of the text (Graneheim and Lundman, 2004; Graneheim et al., 2017). In the present study, sub-categories were grouped into generic categories to reduce the number of categories. Finally, the generic categories were grouped into higher-order categories as main categories. An example of the analysis process is presented in Table 3. The final categories were checked against the initial coding and grouping processes for consistency.
3. In the final phase, reporting, the content of the categories was synthesized as results based on the aim of the study: to explore the core CFI's contribution to identifying the salient existential themes shared by adolescents while receiving treatment in a specialized mental healthcare inpatient unit in Norway. The results are presented in Table 4 and in the results section.

**Table 4:** Main categories and generic categories.

**Main categories**	**Category 1 striving to achieve a normal everyday life**	**Category 2 seeking supportive understanding from family and clinicians**	**Category 3 dealing with adverse childhood experiences**	**Category 4 struggling with ongoing challenges and future uncertainty**
Generic categories	Need for structure Meaning through various activities The importance of relationships	To be heard and seen in treatment Support in treatment Difficulty in talking about problems	Family-related problems from childhood Experiences from school Responsibility for siblings	Experiencing physiological and emotional strain Battling with self-image Struggling to be hopeful

### Ethical considerations

The study was conducted in accordance with the Helsinki Declaration (World Medical Association, 2013), which covers research on vulnerable patient groups. The study protocol was approved by the South-Eastern Regional Committee for Medical and Health Research Ethics (REK SørØst) (2019/317-3) and reported to the Data Inspectorate in Innlandet Hospital Trust (Case No. 109076). The core CFI is a validated tool (Lewis-Fernandez et al., 2017). Moreover, there have been no reports of harm from the patient's perspective following the completion of the CFI (Jarvis et al., 2020). All consent forms followed the rules for anonymity, storage of data, and possible advantages and disadvantages that apply to medical research. All participants gave written informed consent. Participants under the age of 16 years and their caregivers were asked to give their written consent for participation in the study by the responsible clinician at the inpatient unit. Participants 16 years and older were able to provide consent without additional signed consent from their caregiver. However, the caregivers were still informed about the participation. Due to the potentially sensitive nature of the core CFI, the participants had available support from their responsible clinician after the interview.

## Results

The results are illustrated in Table 4, which presents four main categories and three generic categories under each main category. To provide a clear connection between the findings and the core CFI data, the references to the specific CFI questions (Q) are indicated in parentheses after each quote, using the format “(Q.X).”

### Category 1: striving to achieve a normal everyday life

Striving to achieve a normal everyday life was related to the adolescents' need for structure in their lives; they described how life had been turned upside down when they became ill. They expressed a profound sense of loss at being deprived of their everyday routines. This was stated as follows: “My problem is simply that I need to get back to a normal life” (Q.1). The onset of the COVID-19 pandemic exacerbated these feelings, as expressed in the following way: “The pandemic struck, closing the schools, and as the treatment had just begun, it was suddenly stopped. I'm feeling worse than I've ever been” (Q.12). The participants highlighted the importance of having a daily plan and goals, which had been lacking previously.

The participants reported experiencing meaningfulness through various everyday activities such as playing the guitar, playing online video games, listening to music, painting, and drawing. Going for a run, or simply going for a drive, served as strategies for managing anger and frustration. They described finding support in activities that encouraged socialization, such as playing handball. In contrast, lack of energy made their lives profoundly challenging: “I can't function like a normal teenager. There are lots of things I wish I could do, if only I had the energy to cope with them” (Q.3).

The adolescents shared narratives about the importance of relationships in their lives. They mentioned simple moments such as socializing with friends, moments of having fun, and enjoying being together: “A sense of meaning, it's really about the little things, like when you're with friends and you feel like you're having a good time, you just focus on laughing and just having a good time with friends” (Q.8). In this connection, social support was seen as central to a sense of meaningfulness:

What is meaningful for me is having friends and people around me who support me every day. It's reassuring, I have a lot of people who care about me. That's what makes my life meaningful because it kind of comforts me to have someone to turn to, and I have a lot of them (Q.8).

In addition, relationships with animals were mentioned as a positive aspect, such as being with a horse:

Horses give me support because you can say things to a horse, and it won't tell anyone else. You can trust it, and when I'm around horses, I feel quite calm and secure. It's easier to be myself than when I'm around people because I struggle so much then (Q.8).

In the narratives, lacking friends was described as difficult to deal with. The participants expressed a desire to build strong friendships and expand their social network. This was stated as follows: “Maybe get some good friends, maybe get to know someone” (Q.14).

### Category 2: seeking supportive understanding from family and clinicians

A common aspect of the participants' lives was seeking supportive understanding from their family and from clinicians. It was seen as important that others understood the content of their problems. They found it frustrating to be misunderstood: “Because I'm not my diagnosis, I'm me too” (Q.11). The seriousness of feeling misunderstood was expressed as a kind of punishment, rendering feelings of meaninglessness: “There might be things that make me angry because I feel I'm being misunderstood. I feel like I'm just being punished. That's when my thoughts start to feel meaningless” (Q.8). In situations when clinicians did not listen or ask questions, the narratives contained experiences of receiving the wrong kind of help. This could imply that the help had stagnated and was no longer useful. An incident of being ignored and overlooked by family members and clinicians was explained as follows: “I feel like I've gotten the wrong help, or that people don't quite understand what it's all about. They assume or they think they know why. No one's asked me about it” (Q.13).

Most participants described receiving support in treatment from family members or clinicians. This support involved the true essence of their problems being recognized by people they trusted. There were also experiences of frustration at discrepancies between what they desired and what their parents believed was best in terms of treatment, as expressed in the following quote: “They believe they can just force me to be here” (Q.15). One participant was frustrated about clinicians who repeatedly reminded her about the importance of nutrition and food intake on a daily basis:

I know I need food, but it's not that simple. You can't just tell someone who's struggling with an eating disorder to start eating. Or tell someone who's dealing with depression to just be happy. Or someone with anxiety to not be afraid. It doesn't work that way (Q.7).

This frustration could lead to a negative circle: “If I get angry, I often feel sad afterwards because I feel mean. It always ends in hurting myself. It just gets worse and worse for me” (Q.7).

The participants also described the difficulty of talking about their problems, as seen in the following quote: “I don't really like talking to people about how I feel. There's no one I have deep conversations with” (Q.5). In this connection, they mentioned that only their parents and maybe their grandparents were aware of their problems. This situation had made them feel isolated. Treatment sessions involving both the adolescents and their family members were emphasized as problematic as the family members tended to speak on the adolescent's behalf or interrupt when the adolescent tried to say something. One-on-one sessions with clinicians were therefore preferred.

### Category 3: dealing with adverse childhood experiences

The participants shared experiences of dealing with adverse childhood experiences. These involved family problems such as the absence of a parent, death in the family, unresolved family conflicts, and addiction and health problems among family members. This was summed up as follows: “I've experienced a lot of pain from a very young age” (Q.4). They found it difficult to live with divorced parents due to regular moves between two homes. One narrative involved dissatisfaction with the family's living conditions due to hygiene problems. This led to reduced appetite at meals and embarrassment when having visitors. There were also concerns about family members' mental health: “I often had to learn things on my own. I haven't received much help with things” (Q.9). Experiences of excessive alcohol consumption were highlighted as affecting the participants' sense of security in family gatherings: “At a very young age, I always dreaded New Year's Eve and Christmas Eve. And as I got older, I could dread social settings if we were expecting guests, because I knew there would be alcohol” (Q.8).

Other childhood narratives were related to school experiences, particularly bullying from classmates and problematic relationships with teachers. In the former case, the bullying had meant changing schools: “I was bullied from the start of third grade and we eventually had to move because it got so bad. We moved when I started fifth grade, but by sixth grade, the bullying started again” (Q.9). The participants' relations with teachers at school were sometimes underlined as problematic:

I was incredibly scared about something, or I was sad, and then the teacher scolded me because I felt like that. Teachers have never listened to me, they've never supported me in any way. I don't think any elementary school teacher trusts what a fifth grader is saying (Q.9).

The participants described feeling responsible for their brothers and sisters. It was painful to witness their siblings' distress, especially when their own struggles had become a burden. In one case, this sense of responsibility was due to a younger sister's quality of life:

No one should be mean to her, and I can give her good advice that will strengthen her mentally, how to cope with it. This has become incredibly important over the last year, the fact that I've felt this responsibility (Q.8).

The participants also presented positive narratives of family memories from their childhood, such as visiting grandparents or other relatives: “It's fresh air, it's freedom in nature, and it's really just good to get away” (Q.8). These narratives also involved family members such as their mother who had provided a sense of security during their childhood.

### Category 4: struggling with ongoing challenges and future uncertainty

The participants' narratives concerned ongoing challenges they struggled with in their daily lives, coupled with a sense of uncertainty about the future. This was expressed as “I feel like I'm not getting anywhere in life” (Q.8). They described their daily struggles in terms of physiological and emotional strain. Problems with food, weight, and self-esteem dominated their lives: “These problems take a lot of thought and time. They make me feel less happy than I used to be” (Q.3). Another narrative concerned problems with anxiety, depression and lack of energy, and was summarized in this way: “I'd describe my problems as quite painful, in fact” (Q.1). Social anxiety problems combined with low school motivation contributed to a sense of meaninglessness. These problems led to constant pressure and headaches, and difficulty in functioning. This was formulated as “my body shutting down” (Q.3).

The participants described a battle with their self-esteem in social interactions: “I struggle a bit with friends, which makes me doubt myself. When I start overthinking, I end up feeling there's something wrong with me” (Q.4). In another narrative, the treatment was related to expectations from others that were difficult to fulfill: “So it's like a painful place to be, always disappointing other people, or feeling like you disappoint others or yourself. And you're not happy no matter what” (Q.8).

The participants underlined how they found it difficult to be hopeful about the future, which affected any plans they made: “I find it kind of hard to think about the future, so it's not really something I think about. I'm also not a fan of planning. It's more like I'm just trying to get through the week” (Q.14). This struggle was also linked to uncertainty about whether the treatment would be helpful, with some expressing doubt about its effectiveness. The possibility of involuntary hospitalization was also part of their worries about the future: “My family just want me to get better through treatment, so I know they've talked about involuntary admission” (Q.15).

## Discussion

This study aimed to explore the core CFI's contribution to identifying the salient existential themes shared by adolescents while receiving treatment in a specialized mental healthcare inpatient unit in Norway. An inductive content analysis of six core CFI semi-structured interviews resulted in four main categories of importance for the adolescents: (1) Striving to achieve a normal everyday life, (2) Seeking supportive understanding from family and clinicians, (3) Dealing with adverse childhood experiences, and (4) Struggling with ongoing challenges and future uncertainty. On this basis, the four main categories of the present study will be further discussed one by one.

In the first category, *Striving to achieve a normal everyday life*, the adolescents expressed a strong need to return to normalcy, finding their situation to be an overwhelming disruption in their lives. Their narratives highlight how hospitalization due to mental health problems disrupted normal routines and social connections, leaving them with a deep sense of loss by removing them from their everyday routines and meaningful social connections with friends, family and animals. This disruption extended beyond practical concerns to touch on deeper existential questions about meaning and belonging. For example, the loss of routines and relationships challenged their sense of identity and purpose, underscoring the importance of structure in creating a foundation for meaning-making. However, the participants also shared health narratives about health-promoting coping strategies and resources in line with the understanding of narratives by Kirmayer et al. (2023). For example, activities such as music and sports helped them to manage emotional challenges and find meaning in daily life at the unit. The content of this category can be understood as expressions of the existential dimension in line with DeMarinis' definition (2006, 2008), highlighting the importance of daily routines for providing structure and for indicating what is taking place in relation to both aspects of suffering as well as resilience. This category supports the recommendation of Lloyd et al. (2016) to address existential concerns in the everyday experiences of young women in mental healthcare to strengthen their resources and future expectations.

In the second category, *Seeking supportive understanding from family and clinicians*, the participants emphasized the importance of being seen by the clinicians as more than their diagnoses. The narratives revealed that the core CFI provided information about the social dimension, particularly the impact of relationships on wellbeing. In addition, the narratives addressed how lack of genuine support intensified mental health challenges and feelings of isolation and meaninglessness. This desire to be seen and understood reflects a deeper existential need for recognition and validation as whole individuals (Garnow et al., 2022, 2024a,b). Adolescents' struggles with being reduced to their diagnoses highlight the importance of addressing existential concerns about identity and relational meaning in treatment. Research on the existential dimension in young woman in mental healthcare treatment, finds that a safe and relaxed family context is linked to experiences of meaningfulness (Lloyd et al., 2016), thus highlighting the importance of integrating social and existential dimensions into treatment.

The potential of the core CFI in eliciting social determinants of mental health has been explored in research (Aggarwal et al., 2023). The impact of supportive understanding in mental healthcare treatment for adolescents is consistent with findings from PCC research (Morgan and Yoder, 2012; World Health Organization, 2012; Svamo et al., 2024), which highlight the significance of the therapeutic alliance. This is supported by the Norwegian qualitative study by Stige et al. (2021), which explored adolescents' experiences of entering therapy initiated by others. The study shows how adolescents often remained in therapy out of obligation rather than personal engagement. Positive therapeutic experiences are linked to clinicians who are flexible, genuine, and able to connect on a personal level. Importantly, adolescents described feeling responsible for making therapy work, highlighting the need for flexible, individualized approaches that make room for adolescents' own voices (Stige et al., 2021). Likewise, Cirasola et al. (2024) underline how ruptures in the therapeutic alliance and epistemic mistrust may lead to disengagement, and how restoring trust requires therapeutic sensitivity and tailored communication. These findings support the value of tools like the core CFI in facilitating engagement and helping clinicians better understand and respond to adolescents' unique concerns. Findings from prior studies about PCC and help-seeking behaviors (Radez et al., 2021; Westberg et al., 2022; Svamo et al., 2024) highlight how themes concerning isolation, self-reliance, and complex family relationships affect adolescents' mental health. As in the studies that explored the use of the core CFI with Ethiopian adolescents with eating disorders in Israel (Shem-Tov et al., 2018) and with parents in family-based mental healthcare (Sanchez et al., 2022a,b), these results show the importance of understanding the cultural and familial context in mental healthcare by placing value on the patient's preferences.

The third category, *Dealing with adverse childhood experiences*, revealed the impact of early-life experiences of suffering on shaping present struggles in adolescents' mental health. The participants described challenges such as parental absence, family conflicts, addiction, and difficult living conditions, which created a sense of emotional distress. Conversely, positive experiences like visiting grandparents offered moments of stability and joy. These narratives highlight how adverse childhood experiences contributed to existential struggles, such as searching for meaning, coherence, and belonging, which are fundamental to their sense of identity and wellbeing. The CFI facilitated a process whereby the adolescents reflected on their life stories. The CFI questions related to background, identity and meaning (Questions 8–10) were important for exploring their own cultural ways of handling existential concerns in terms of making decisions and meeting challenges in everyday situations (DeMarinis, 2003). In line with DeMarinis' research (2006, 2008), the participants' accounts revealed a search for meaning, belonging, and coherence across time, highlighting the existential dimension of suffering as it relates to identity, responsibility, and hope. Kirmayer and colleagues (Kirmayer et al., 2023) emphasize how self-understanding framed through metaphors such as “experiencing a lot of pain from childhood” shapes symptom interpretation, help-seeking behavior, and treatment responses. Based on Kleinman's understanding of illness narratives, the narratives in this category added coherence to distinctive life events and their long-term course of suffering (Kleinman, 1988). These findings align with previous research on using the core CFI with adults (Aggarwal et al., 2015; Muralidharan et al., 2017), which emphasizes that its use as a narrative tool enhances a person-centered approach by bridging cultural, social, and psychological dimensions.

The fourth category, *Struggling with ongoing challenges and future uncertainty*, concerned narratives about mental and physical health problems and uncertainty about the future. The adolescents described feelings of being stuck, doubting the efficacy of their treatment, and wondering if they would ever get a better life. Their suffering was not only rooted in current symptoms but also in a broader existential struggle regarding agency, purpose, and the passage of time. This highlights the importance of including and exploring the interactions between the psychological and the existential dimension in mental healthcare for adolescents (Olstad et al., 2022, 2023). in line with research in this area. The Norwegian study involving therapists working in a mental health outpatient clinic for adolescents (Ulland and DeMarinis, 2014), underlines that addressing fears, loneliness, and the search for meaning can alleviate distress and enhance wellbeing. The narratives in this category emphasized the interconnection among the dimensions in Kleinman's original cultural and multi-dimensional model of a healthcare system (Kleinman and Benson, 2006; Kleinman, 2016) as well as in the adapted Scandinavian model by DeMarinis (2003, 2008). The narratives framed symptoms and suffering within a broader life context, helping patients make sense of their experiences and guiding how they seek help and respond to treatment (Kirmayer et al., 2023). By framing symptoms and suffering within a broader life context, the CFI helped adolescents articulate their struggles in ways that connected their immediate challenges to larger existential concerns.

The four main categories in the present study demonstrate that the core CFI contributed to identifying the salient existential themes shared by adolescents while receiving treatment in a specialized mental healthcare inpatient unit in Norway. Among these dimensions, the existential dimension emerged as particularly central, shaping how adolescents interpreted their symptoms, engaged with treatment, and sought meaning in their experiences. The findings highlight the importance of including a clinically-applicable, multi-dimensional model for healthcare that includes existential dimension in treatment for and with adolescents for three reasons. Firstly, the existential dimension is seen as central for delivering PCC as it informs what it means to prioritize the unique needs and preferences of the person seeking help in a holistic way (Morgan and Yoder, 2012). The existential dimension is underutilized in PCC research, and is often limited to traditional religious expressions of meaning. A clinically-applicable multi-dimensional model that includes the existential dimension, understands this dimension as being relevant for use with all patients, just as the CFI is intended for use with all patients. Secondly, the existential dimension plays a fundamental role in both shaping and influencing changes relevant for the other dimensions (biological-physical, psychological, social, and ecological), and assisting with identifying sources of distress and hope of importance for all of the dimensions (DeMarinis, 2003, 2006, 2008). In this way, the core CFI not only identifies distinct dimensions, but also reveals how the existential dimension functions as a dynamic force that shapes and connects the dimensions. More specifically, the core CFI contributes to structuring clinical conversations to capture adolescents' personal meanings and experiences across the different dimensions of health. These findings concur with the holistic, multi-dimensional approach to illness and health (Kleinman, 1980; DeMarinis, 2003). Hence, this knowledge may contribute to a better understanding of the therapeutic process. However, further research is needed to explore how these factors influence the therapeutic process over time. Thirdly, including the existential dimension in treatment for adolescents is important for addressing vulnerabilities such as emotional regulation, relational loss, and existential fears through strategies informed by the adolescents' narratives (Ulland and DeMarinis, 2014; Lloyd et al., 2016). Responses to the added CFI sub-questions in item 8 (see Table 2), about what gives life meaning and what makes it feel empty or meaningless, highlighted how existential concerns can be articulated in relational and everyday terms.

The adolescents in the present study came from a wide geographical area close to nature. The broad and dynamic understanding of culture in the DSM-5 (American Psychiatric Association, 2013, 2022), which underpins the core CFI, functioned well as a framework to capture adolescents' diverse backgrounds, living conditions, practices, and values. It contributed to contextualizing their experiences of physiological, emotional, and existential strain within a broader cultural and social framework. Thus, the study supports the value of the core CFI in bringing cultural psychiatry and medical anthropology into clinical practice (Kleinman, 2016).

### Clinical and research implications

Mental health problems in adolescents are the leading health burden in this age group worldwide, thus representing a significant social concern that places a burden on individuals, families, and society (World Health Organization, 2018, 2024). The situation is critical as many adolescents do not receive the support they need from the healthcare system (Radez et al., 2021; Westberg et al., 2022). The World Health Organization emphasizes the importance of integrating adolescents' needs, wishes, and preferences into their mental health treatment (World Health Organization, 2018, 2024) and PCC is seen as essential in addressing these concerns (World Health Organization, 2012). From the perspective of adolescents seeking help in mental healthcare, self engagement in treatment has been highlighted as central in PCC (Morgan and Yoder, 2012; Svamo et al., 2024). In this connection, the DSM-5 core CFI serves as a tool to strengthen PCC in clinical settings by eliciting patients' narratives of illness and health (Kirmayer et al., 2023). According to Kirmayer et al. (2023), narratives influence symptom interpretation, help seeking and treatment response. The central findings in this study were based on analysis of these types of narratives, as told through the cumulative responses to the CFI questions, which were shaped by daily interactions and shifting personal circumstances (American Psychiatric Association, 2022). Furthermore, the narratives concerned a multi-dimensional understanding of illness and health, grounded in what was important for these adolescents in mental healthcare treatment. This was related to the functional understanding of DeMarinis' approach to the existential dimension (DeMarinis, 2003, 2006, 2008), based on Kleinman's cultural and multi-dimensional healthcare model and the central role of the symbolic dimension (Kleinman, 1980). The existential dimension has been used as an umbrella term to cover a broad range of expressions of meaning-making, anchored in the cultural context and everyday life of the individual (DeMarinis, 2003, 2006, 2008).

The results in the present study suggest how the core CFI can contribute to competence-building in specialized mental healthcare by facilitating a structured, person-centered approach. In adapting the core CFI to a specialized mental healthcare context in Norway, each question was carefully reviewed and adjusted in collaboration with clinicians. This included training inspired by Aggarwal and colleagues (Aggarwal et al., 2016b), emphasizing the need for contextual adaptation and cultural sensitivity in the specific clinical setting. The adaptation aligns with the central question posed by the Norwegian Institute of Public Health: “What is important to you?” (Norwegian Institute of Public Health, 2024). This question is seen as fundamental in the National Patient Pathway [Helsedirektoratet (Norwegian Directorate of Health), 2018] to increase mental health service user involvement and encourages meaningful conversations between adolescents, families, and healthcare providers, supporting a person-centered approach. The core CFI has the potential to support this aim by enabling clinicians to elicit what truly matters to adolescents, especially regarding existential concerns.

Clinically, there is a need for comprehensive CFI training for clinicians to understand the purpose of the core CFI and increase adherence to the CFI script (Aggarwal et al., 2014; Muralidharan et al., 2017). Previous studies (Aggarwal et al., 2016b) indicate that without sufficient training, clinicians may struggle to apply the core CFI meaningfully. The study from the Netherlands (Silvius et al., 2024) showed that a lack of mandatory training limited adoption and led to perceptions of the CFI as burdensome. In a Norwegian outpatient context, clinicians have reported uncertainty about addressing existential topics with adolescents (Ulland and DeMarinis, 2014), highlighting a clear need for strengthened clinical competence in this area, and the use of a clinically-applicable approach to the existential dimension. This study shows that the core CFI, particularly through the sub-questions “What gives you most meaning in life?” and “What makes you experience life as empty or meaningless?” activates entry into the existential dimension, which, according to DeMarinis (2003), is not an isolated dimension but one that influences and connects biological, psychological, social, and ecological aspects of health. Thus, the core CFI may serve as a competence-enhancing tool, enabling clinicians to recognize, explore, and respond to existential topics in a therapeutic and clinically-appropriate way.

The findings support the relevance of integrating the existential dimension in studies done in a variety of clinical contexts (DeMarinis, 2003; Haug et al., 2015, 2016; Lilja et al., 2016; Vattø et al., 2020; Søberg et al., 2022), highlighting its importance in addressing individual needs and enhancing PCC. There is a growing call for more research to investigate the applicability of the core CFI to younger populations (Kirmayer, 2015; Rousseau and Guzder, 2016; Lewis-Fernández et al., 2020; Jones-Lavallée et al., 2022), and this study contributes to filling this gap. Further research is needed in two key areas. First, more studies should examine the broader application of the core CFI in adolescent mental healthcare, including how it can be adapted to better fit this population's unique needs. Second, the role of the existential dimension in mental healthcare in general requires deeper exploration to determine how it can be systematically integrated into clinical practice. Future research should examine how existential concerns influence the mental health of adolescents. Such studies should also examine how clinicians can identify and engage effectively with existential issues in a structured and therapeutic way to deepen understanding of mental health patients' concerns, symptoms and resources and to plan appropriate treatment strategies.

### Rigor and trustworthiness

This study used the core CFI and followed the CFI process throughout the treatment period with six consecutive adolescents with severe mental disorders admitted to a specialized mental healthcare inpatient unit in Norway. The core CFI was used effectively, eliciting rich narratives from the adolescents about what they found important while receiving treatment, despite the severity of their conditions and the long-term nature of their admission.

To ensure transcription accuracy and cultural appropriateness, transcripts were carefully reviewed and cross-checked with co-authors experienced in Norwegian mental health settings. For publication, selected quotes were translated into English to preserve their meaning and context. The translations were reviewed by a professional language editor fluent in both Norwegian and English, with a deep understanding of Norwegian culture. Any discrepancies were resolved through collaborative discussion among the authors.

The analytical strategy used was inductive content analysis, allowing for in-depth exploration of the latent content of the narratives. The strength of this strategy lies in its flexibility and ability to uncover underlying meanings in qualitative data, providing nuanced understandings of participants' experiences. Given the complexity of the adolescents' perspectives and the lack of previous CFI studies on this population, this approach was chosen to ensure that detailed insights from the adolescents were taken into consideration. The credibility of the analysis was further strengthened by collaboration between the authors at every stage of the analysis. This collaboration ensured a balanced and comprehensive analysis. The iterative nature of the analysis process ensured that the final categories reflected not only the participants' narratives but also the dynamic structure of the CFI itself. This approach highlights how the CFI facilitated the elicitation of narratives and provided a framework for understanding the adolescents' experiences. To strengthen the trustworthiness of the study, the authors discussed the interpretations of the findings broadly and consistently, which is expected to reduce the influence of researcher biases (Graneheim et al., 2017).

Although CFI trained and experienced clinicians administered the core CFI, the researchers were aware of the potential for bias in the sample selection. The first author's familiarity with the unit enabled close collaboration with the clinicians, ensuring continuity and rapport in the data collection process. However, conducting research at one's own workplace can pose challenges. The potential for bias existed due to the first author's insider position, as familiarity with the clinical context might have influenced the interpretations. To counter this, the co-authors collaborated closely in the process. The co-authors bring multidisciplinary competencies, including clinical psychology, mental health expertise and clinical care. Notably, mental health expertise helped maintain methodological rigors related to accurately supporting the study's findings especially the mental health categories. External perspectives and extensive expertise in CFI research on the part of the co-authors enhanced the robustness of the analysis and helped to reduce bias.

### Limitations

This qualitative study did not include a control group, which is a clear limitation. However, as far as possible, similar patient files were reviewed by the clinical staff and notes were made on differences or nuances in information that emerged through the CFI process relevant to treatment planning and degree of adolescent engagement. Another limitation of this study is the small sample size, which consisted of six consecutive patients. The restricted number is due to the methodological format of the larger mix-methods project which the present study is a part of. The design concerned efficacy testing of the CFI, including patient interviews at three different points of time. The results from this CFI process are included in another study. A third limitation is that no gender reflection can be done to the consecutive patient inclusion of five females and one male in the study. The limited number of male participants restricts the generalizability of findings to male adolescents, and we recommend future studies with a larger, more gender-balanced sample. The initial testing of the core CFI with three adolescents aged 14–15 before broader implementation ensured the suitability of the core CFI and allowed for necessary adaptations in collaboration with clinicians. This proved to be highly relevant, as five of the six participants were under 16 years old.

### Conclusion

This study highlights the potential of the core CFI as a narrative tool to enhance understanding of adolescents' needs in mental healthcare treatment, supporting its inclusion in the National Patient Pathway for adolescents in mental healthcare in Norway (2018). Through the core CFI, adolescents articulated multi-dimensional narratives related to illness and health, encompassing biological-physical, psychological, social, ecological, and existential dimensions. Among these dimensions, the existential dimension emerged as particularly central, shaping how adolescents reflect on their experiences, interpret their symptoms, and engage with treatment. Their narratives emphasized the need for normalcy, supportive relationships, and existential concerns in relation to past and present challenges, as well as uncertainty about the future. The existential dimension was explored from a clinically-applicable, functional perspective, focusing on the role or purpose for adolescents in making decisions and meeting challenges in everyday situations. It appeared to serve a central function by providing a deeper and more nuanced view of the other dimensions. These findings underscore the importance of acknowledging existential concerns as an integral part of adolescents' experiences and incorporating them into treatment to deliver PCC. The ability of the CFI to elicit these themes demonstrates its value in enabling clinicians to explore existential topics in a structured and therapeutic manner. Further research is needed to explore the broader application of the core CFI in mental healthcare treatment for adolescents and to further examine how existential concerns shape mental health outcomes. Investigating how clinicians can meaningfully engage with these topics will be essential for refining clinical approaches. Additionally, adapting the core CFI to better address the unique needs of adolescents will contribute to the development of structured strategies to improve mental health support. This includes refining sub-questions related to existential themes such as identity, meaning, and future orientation, to better align with the developmental needs as well as the particular cultural context's resources and challenges of adolescents.

## Data Availability

The raw data supporting the conclusions of this article will be made available by the authors, without undue reservation.
