# Supplementary material for: “I need to get back to a normal life”: using the core DSM-5 Cultural Formulation Interview to explore existential themes shared by adolescents in specialized mental healthcare in Norway
Source: Front Psychol. 2025 Nov 12;16:1652189. doi: 10.3389/fpsyg.2025.1652189 (PMC12646933; doi:10.3389/fpsyg.2025.1652189)
Supplement: Supplementary file 1 [file Data_Sheet_1.pdf]

Supplementary file

Article title: *Explanations for the rationale for adaptions to the DSM-5 core CFI for use in an adolescent mental health clinic in Norway*

1. Additional words and phrases:

- Question 6: the phrase “social media” was added to reflect the role of social media in relation to support.
- Question 7: the word “school” was added to highlight the potential influence of the school environment in exacerbating the problem.
- Question 12: the word “friends” was added as a possible source of help.
- Question 13: the terms “bullying”, “challenges in your family” and “various support services that have not understood you” were added to describe factors that may prevent access to care and support.

2. Additional sub-questions:

- Question 3 “What troubles you most about your problem?”: the sub-question “How do you feel it in your body?” was added because the clinicians expressed the desire to incorporate the participants’ reflections on bodily experiences, considering this is a pivotal and intricate aspect for adolescents.
- Question 8 “What are the most important aspects of your background”: the sub-questions “What gives you most meaning in life?” and “What makes you experience life as empty or meaningless?” were added to make more explicit the implicit existential dimension of the CFI through the inclusion of questions related to meaning in life. The clinicians agreed with the importance of these questions.
- Question 12 “What kinds of treatment, help, advice, or healing have you sought for your [PROBLEM]?”: the sub-question “What kind of help have you sought elsewhere, such as at school, from friends, relatives, leaders, and social media?” was added because the clinicians wanted to explore help-seeking from various external sources.
